# Supplementary material for: Dual role of E-cadherin in the regulation of invasive collective migration of mammary carcinoma cells
Source: Sci Rep. 2018 Mar 21;8:4986. doi: 10.1038/s41598-018-22940-3 (PMC5862898; doi:10.1038/s41598-018-22940-3)
Supplement: Supplementary file 1 — supplementary data [file 41598_2018_22940_MOESM1_ESM.pdf]

Dual role of E-cadherin in the regulation of invasive collective migration of mammary carcinoma cells

Yair Elisha[1], Vyacheslav Kalchenko[2], Yuri Kuznetsov[2], and Benjamin Geiger[1]*

[1]Department of Molecular Cell Biology, Weizmann Institute of Science, Rehovot 7610001, Israel

[2]Department of Veterinary Resources, Weizmann Institute of Science, Rehovot 7610001, Israel

*Correspondence:
B. Geiger
Tel: +972-8-9343910
Mob: +974-52-3488848
Email: benny.geiger@weizmann.ac.il

Disclosure of Potential Conflict of Interest: The authors declare that there are no potential conflicts of interest.

Supplementary information

Diversity of collective migration modes

To verify which cell lines migrate collectively, we used the “nearest neighbor” methodology, in which we seed cells sparsely, grow them to about 20% confluence, then fix them and stain their nuclei with DAPI. In each cell line, we measured the average distance between each cell nucleus, and its three closest neighbors (Fig. S1B). By plotting the percentage of cells against the average three “nearest neighbors” distances, we noticed that 4T1, MCF10A, and MDCK cells create sharp peaks between 20-50 µm. This finding indicates that these three cell lines create cell clusters in which the distance of one cell from its neighbors is small, while H1299 cells, which migrate in a non-collective manner, were distributed quite homogenously. Notably, less than 2% of 4T1 cells contain more than one nucleus, supporting our contention that the cells migrate collectively. Furthermore, the low cell density in the plate indicates that cell clusters were not created by being plated densely. We also confirmed that the cells were thoroughly suspended and seeded as single cells to minimize initial cell-cell interactions.

Supplementary figures

Fig 3 panel E

Full blotting of Figure 3 panel E:

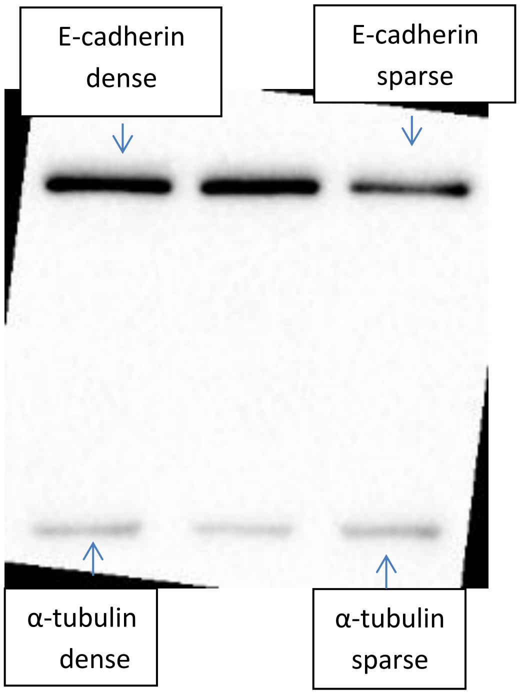

Fig S1

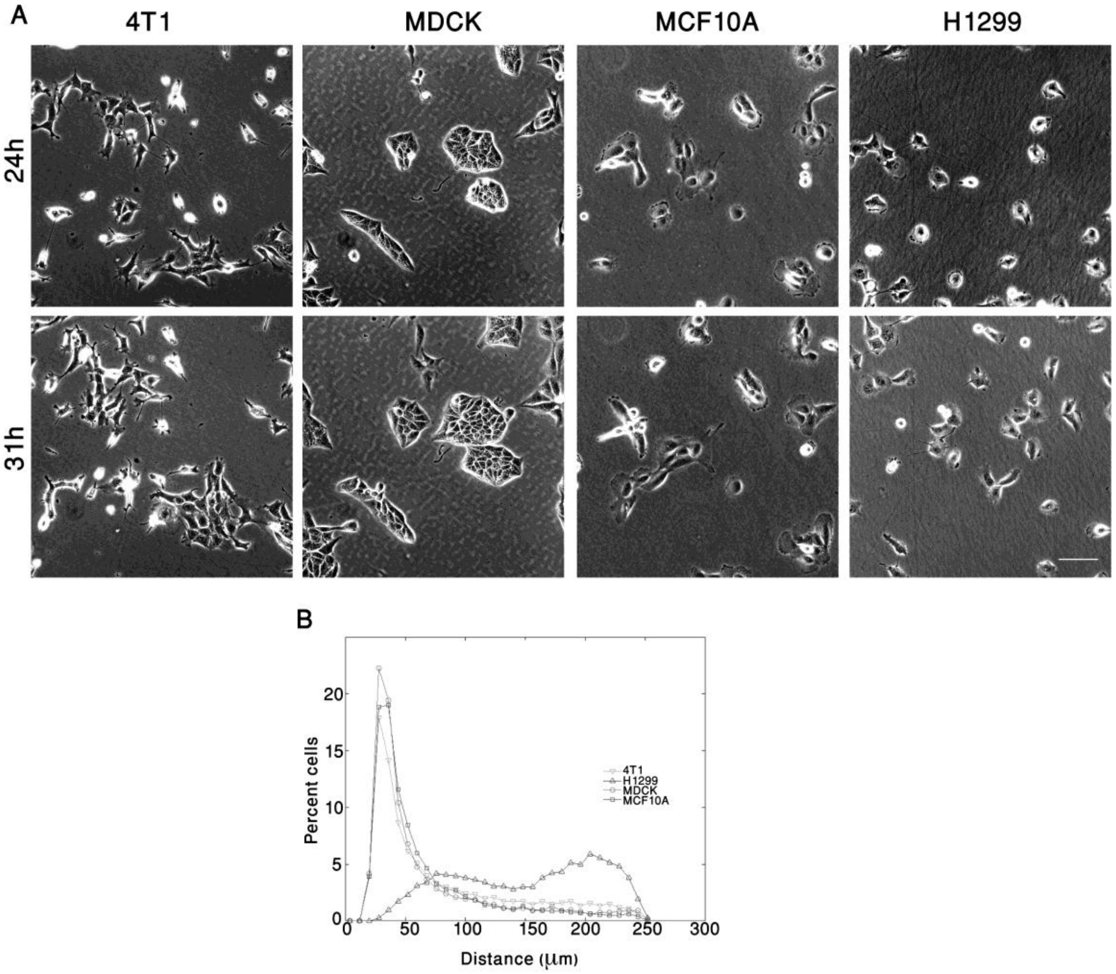
A
4T1
MDCK
MCF10A
H1299
24h
31h
B
Percent cells
Distance (μm)
4T1
H1299
MDCK
MCF10A
0
5
10
15
20
0
50
100
150
200
250
300

Fig S2

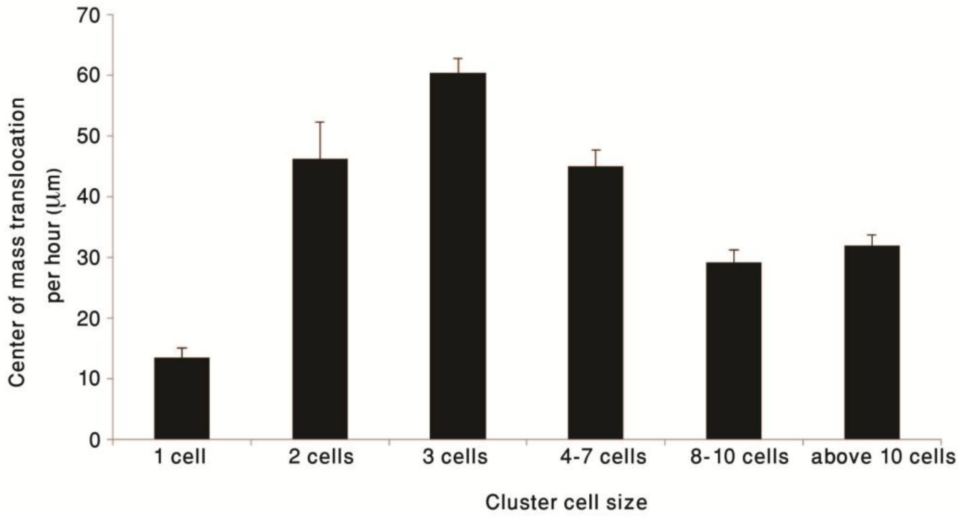

70
60
50
40
30
20
10
0
Center of mass translocation per hour (μm)
1 cell
2 cells
3 cells
4-7 cells
8-10 cells
above 10 cells
Cluster cell size

Fig S3

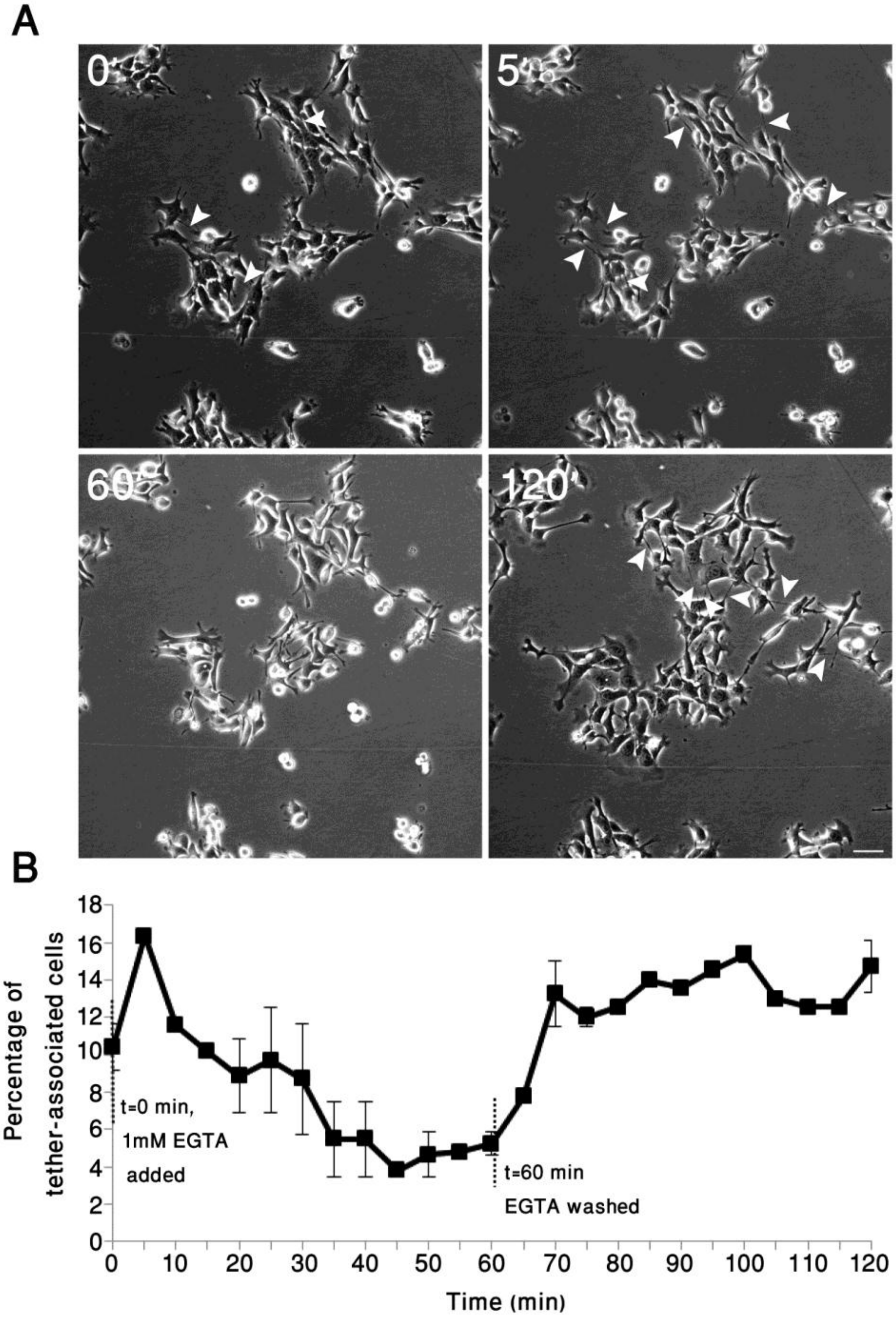
A
0'
5'
60'
120'
B
Percentage of tether-associated cells
0
2
4
6
8
10
12
14
16
18
t=0 min, 1mM EGTA added
t=60 min EGTA washed
0
10
20
30
40
50
60
70
80
90
100
110
120
Time (min)

Fig S4

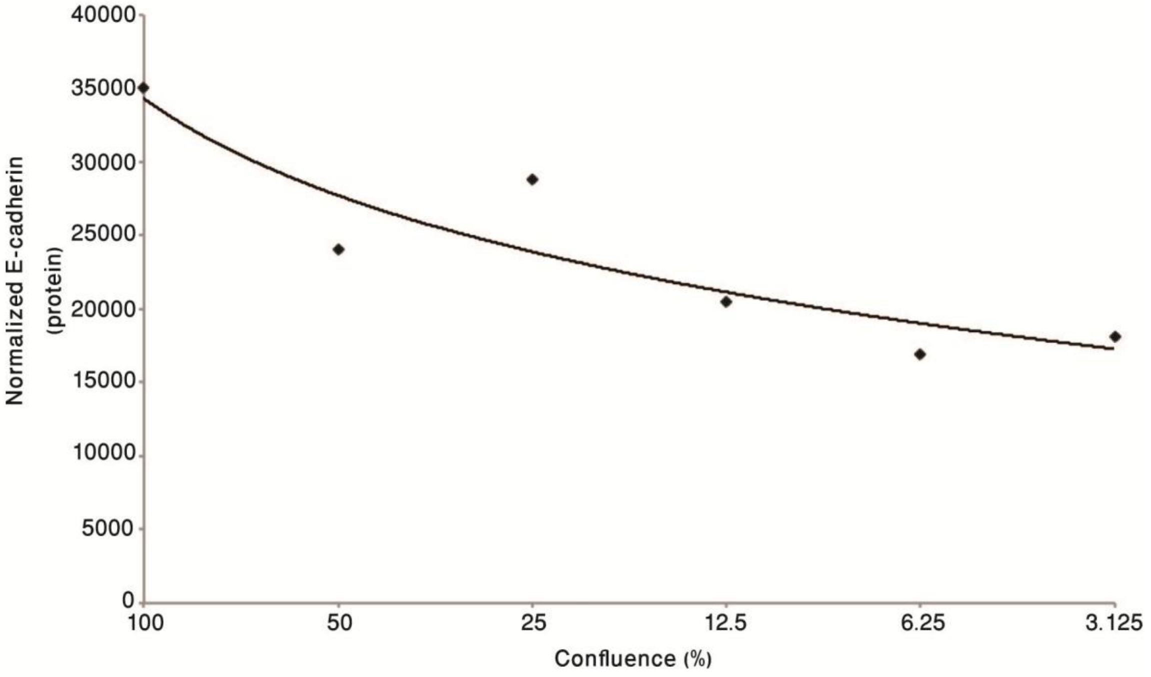
40000
35000
30000
25000
20000
15000
10000
5000
0
Normalized E-cadherin
(protein)
100
50
25
12.5
6.25
3.125
Confluence (%)

Fig S5

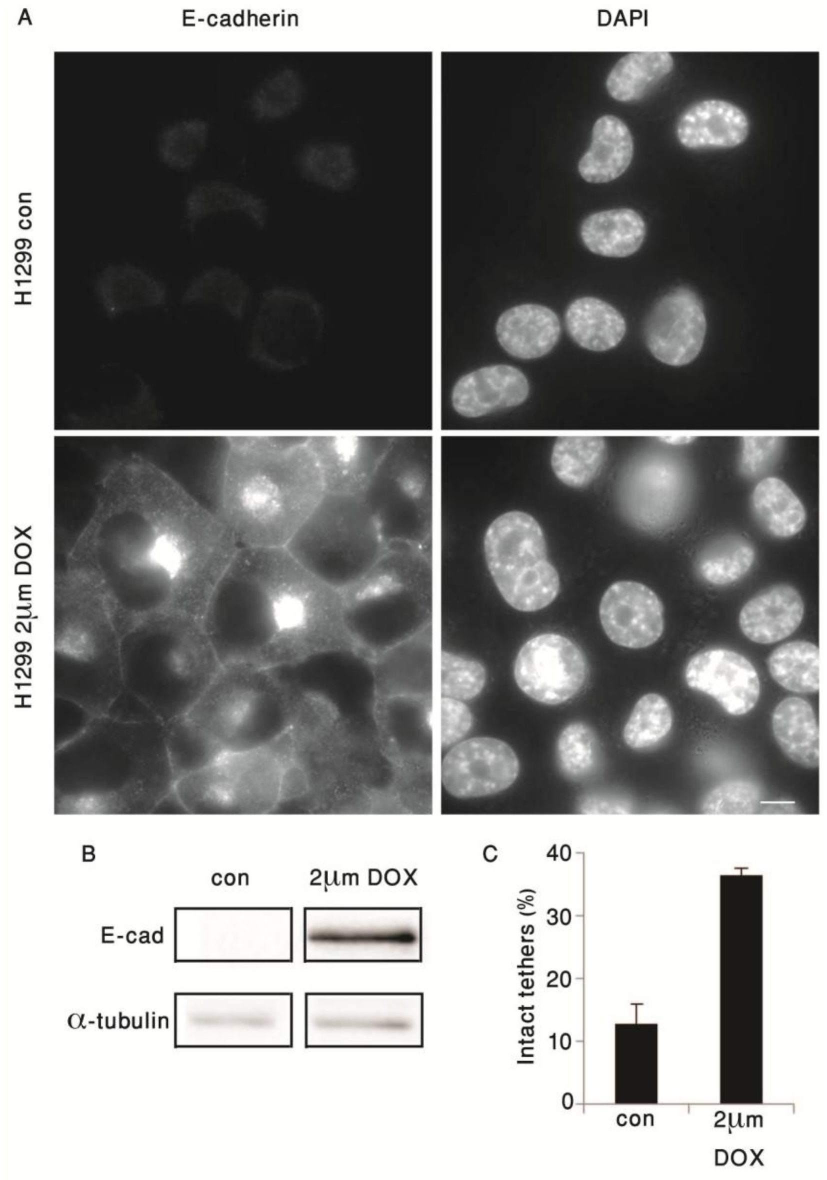
A
E-cadherin
DAPI
H1299 con
H1299 2μm DOX
B
con
2μm DOX
E-cad
α-tubulin
C
Intact tethers (%)
40
30
20
10
0
con
2μm DOX

Fig S6

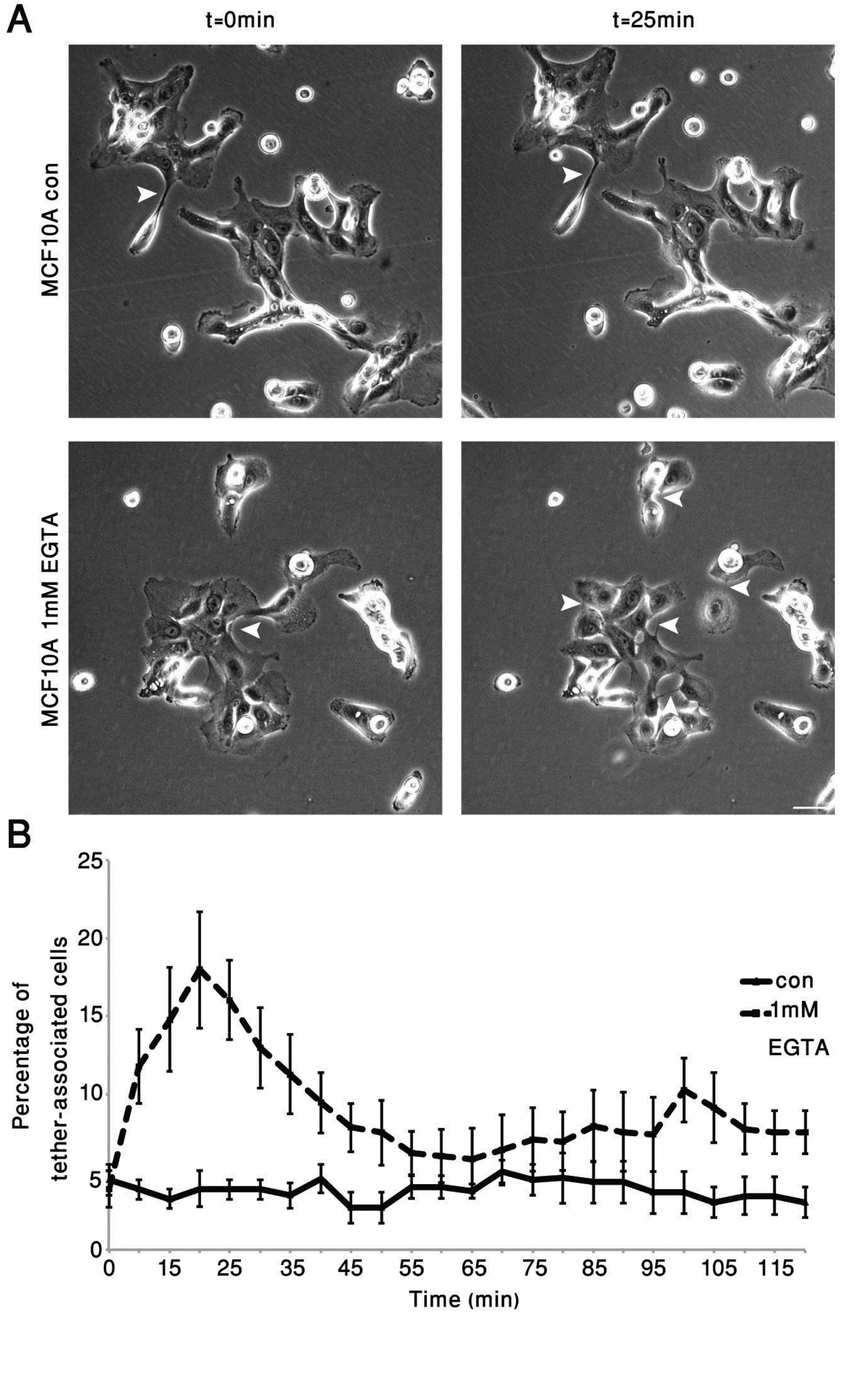
A
t=0min
t=25min
MCF10A con
MCF10A 1mM EGTA
B
Percentage of tether-associated cells
25
20
15
10
5
0
0
15
25
35
45
55
65
75
85
95
105
115
Time (min)
con
1mM EGTA

Fig S7

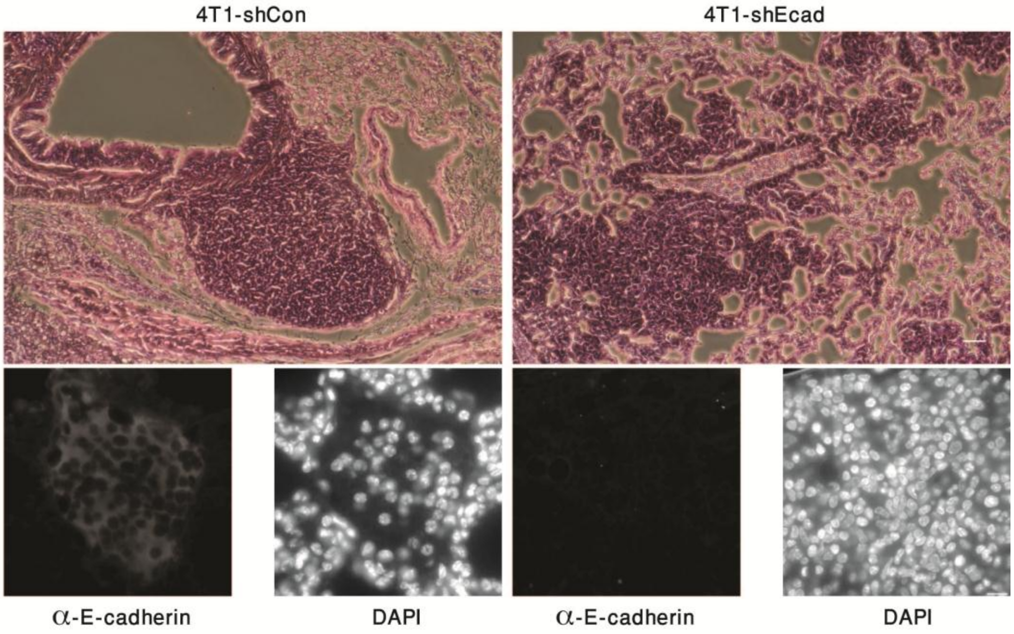

SUPPLEMENTARY FIGURE LEGENDS

Figure S1: Scoring of collective cell migration displayed by different epithelial cell lines, using a "nearest neighbor" collectivity scoring.

(A) Different epithelial cell lines (4T1, H1299, MDCK, MCF10A) were sparsely plated on tissue culture dishes, coated with 10 µg/ml fibronectin (FN) and imaged, using phase-contrast microscopy, 24 hrs and 31 hrs thereafter. Scale bar: 100 µm. (B) For "nearest neighbor" collectivity scoring, the four cell types shown in Panel A, were sparsely plated, and incubated for 24-48 hrs, after which they were fixed and stained with DAPI. The positions of nuclei were automatically recorded, and the average distance between each nucleus and its 3 nearest neighbors calculated. The plot indicates that MCF10A, MDCK and 4T1 maintain sharp peaks, within the range of roughly 20-30 µm between them, while H1299 cells, plated at an identical density, displays a broad peak, ranging from 50-240 µm, characteristic of single-cell migration. Notably, the collective translocation of epithelial islands varies greatly, as can be seen in Supplementary Movie S1.

Figure S2: Small 4T1 cells clusters translocate faster, compared to large clusters or single 4T1 cells.

4T1 cells were cultured for 24 hrs in a tissue culture dishes, coated with 10 µg/ml fibronectin (FN). Cell migration was then monitored by live-cell imaging (5 min interval between frames) . In these movies, we manually measured center-of-mass translocation of different-sized cell clusters over time, using ImageJ software. Each histogram represents the tracking of at least three different-sized cell clusters, in two independent experiments.

Figure S3: 4T1 inter-cellular tethers are calcium-dependent.

4T1 cells were cultured for 24 hrs in tissue culture dishes, coated with 10 µg/ml fibronectin (FN). Cells were imaged just before (t=0), or at different time points after the addition of 1mM EGTA to the medium. (A) EGTA treatment induced cell contraction, leading to the formation of multiple short-lived tethers (t=5 min), which essentially disappeared upon further incubation (t=60min). Replacement of the medium at t=60 min with normal, (Ca^{++}-containing) medium led to gradual increase in tether number, reaching normal levels by 60-120 min. Arrowheads in panel "5 min" and "120 min", point to ruptured tether and newly-formed tether, respectively. Scale bar: 50 µm. (B) Percentage of tether-associated cells was measured at various time points during calcium chelator incubation (0-60 min) and after calcium restoration (60-120 min).

Figure S4: E-cadherin protein levels are affected by plating density of 4T1 cells.
Serial dilution of plated 4T1 cells/cm^2 from $8.9*10^4$ (100% confluence) to $2.8*10^3$ (3.125% confluence) followed by 24 hr incubation, affected E-cadherin levels, as measured by Western blot analysis. Notice that at ~10% confluence or lower, E-cadherin levels display a twofold lower

E-cadherin protein levels, compared to those of cells plated at 100% confluence.

Figure S5: Over-expression of E-cadherin in H1299 cells induces the formation of stable tethers in these cells.

H1299 cells expressing a tetracycline-inducible E-cadherin promotor, were cultured for 24 hrs in tissue culture dish with/without doxycycline (DOX, 2μM). (A) E-cadherin expression was visualized by immunolabeling (A) or Western immunoblotting (B). Notice the increased expression of E cadherin following DOX induction (A and B), and the major increase in tether formation (~2.8 fold) shown in C.

Full blottings of Figure S5 panel B:

Lanes were extracted from the same gel. Exposure time for E-cadherin/α-tubulin were optimized for each protein

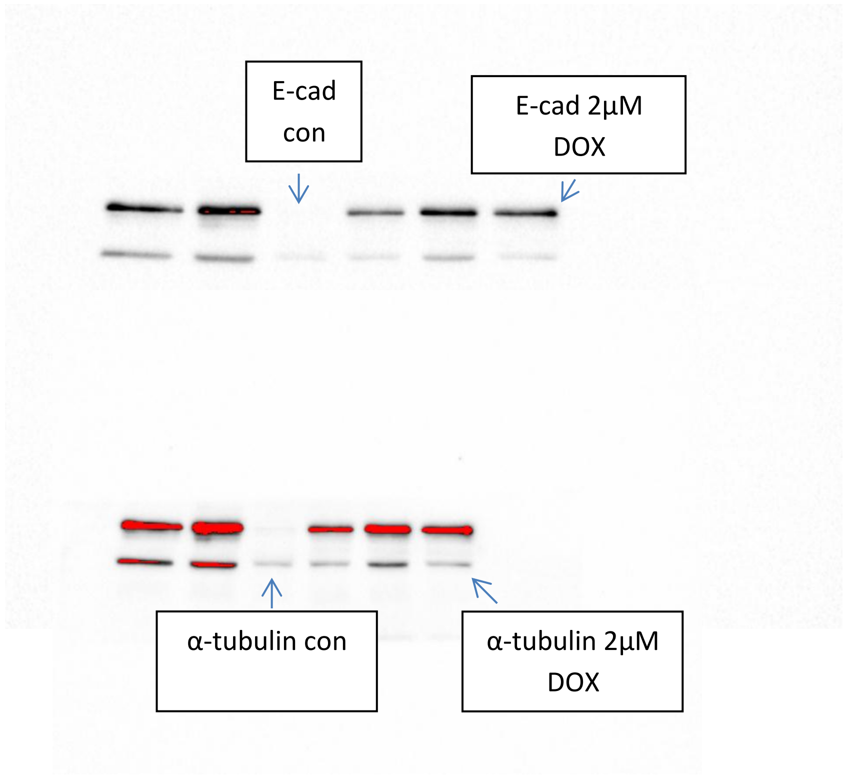

Figure S6: Reduction of calcium levels in the medium, leads to tethers formation by MCF10A cells.

MCF10A cells, which, normally, form robust adherens-type junctions (not mediated via tethers). were plated for 24 hrs in a tissue culture dish coated with 10 µg/ml fibronectin (FN), after which 1mM EGTA was added to the medium (t=0). (A) EGTA treatment induced cell contraction and formation of many short-lived tethers (t=25 min), which essentially disappeared upon further incubation. Arrowheads point to the tethers detected at the indicated timepoints. Scale bar: 50 µm. (B) Percentage of tether-associated cells, measured at various timepoints during EGTA treatment (0-120 min).

Figure S7: Lack of E-cadherin expression persists in lung metastases following injection of 4T1-shEcad cells into the mammary fat pads of BALB/c mice.

Representative histological sections of metastases-containing lungs, examined 14-21 days after fat-pad injection of 4T1-shCon and 4T1-shEcad cells. Immunolabeling demonstrated that while E-cadherin staining is clearly visible in 4T1-shCon cells, no E-cadherin staining is found in 4T1-shEcad mice lung sections. Notice that 4T1-shCon metastasis tended to grow in clusters often located in close proximity to blood vessels, primarily at the lung's periphery, while 4T1-shEcad cells tended to grow in a more disorganized manner throughout the lung. Scale bar: upper images- 100µm , lower images- 50µm.

SUPPLEMENTARY MOVIE LEGENDS

Supplementary Movie S1

Different epithelial cell lines (4T1, H1299, MDCK, MCF10A) were sparsely plated on tissue culture dishes coated with 10 µg/ml fibronectin (FN). Twenty-four hrs post-seeding, cells were imaged, using live cell vide microscopy (with phase-contrast optics; 10x/0.3NA objective; time interval between frames= 5-min) Note the collective mode of migration of MDCK (slow), MCF10A (medium) and 4T1 (fast), and the tendency of H1299 cells to migrate individually. Scale bar: 10 µm.

Supplementary Movie S2

4T1 cells were seeded sparsely on a tissue culture dish, coated with 10 µg/ml fibronectin (FN), and immediately subjected to live-cell imaging,. Cells were imaged every 5 min, using a 10x 0.3NA phase-contrast objective. Scale bar: 10 µm.

Supplementary Movie S3

4T1 cells were seeded on a tissue culture dish coated with 10 µg/ml fibronectin (FN) for 24 hrs, after which the cells were imaged every 5 min for a total of 2.75 hrs, using phase-contrast microscopy with a 10X0.3NA objective. Scale bar: 10 µm.

Supplementary Movie S4

4T1 cells were cultured in 3D collagen gels, as described in Methods. After 16 hrs, the cells were tracked by light microscopy, 200 µm above surface level (inside the gel), every 5 min, for a total of 5 hrs, using phase-contrast video microscopy, using a 20x/0.5NA objective. Scale bar: 50 µm.

Supplementary Movie S5

4T1 cells were cultured for 24 hrs on a tissue culture dish coated with 10 µg/ml fibronectin (FN), after which 1mM EGTA was added to the medium. Arrows point to the tethers tracked during the movie, both prior to and immediately after addition of EGTA. After 60 mins, the medium was replaced by fresh, calcium-containing medium and cells were further imaged for 60 mins with 1 min interval between frames. Live cells imaging was conducted, using phase-contrast microscopy with a 10x 0.3NA objective. Scale bar: 50 µm.

Supplementary Movie S6

H1299 cells expressing a tetracycline-inducible E-cadherin were cultured for 24 hrs in a tissue culture dish with (DOX) or without (H1299 con) 2μM doxycycline. Cells were imaged every 5 minutes for a total of 495 mins, using phase-contrast microscopy with a 10x 0.3NA objective. Scale bar: 50 μm.

Supplementary Movie S7

MCF10A cells were cultured for 24 hrs on a tissue culture dish coated with 10 μg/ml fibronectin (FN). Cells were then subjected to live cell video imaging, and 1mM EGTA was immediately added to the medium (MCF10A, 1mM EGTA). As controls, MCF10A cells, in another plate, were tracked without the addition of EGTA (MCF10A con). Cells, on both plates, were imaged every 5 min for a total of 120 mins, using phase-contrast microscopy with a 10x 0.3NA objective. Scale bar: 50 μm.

Supplementary Movie S8

4T1 shCon and -shEcad cells were seeded, separately, on tissue culture dishes coated with 10 μg/ml fibronectin (FN). Twenty-four hrs post-seeding, cells were imaged every 5 min for 8.25 hrs total, using phase-contrast microscopy with a 10X0.3NA objective. Note the tethers formed by 4T1-shCon cells, which connects them to neighboring cells, and the lack of tether formation from 4T1-shEcad cells. Scale bar: 10 μm.

Supplementary Movie S9

4T1-GFP-shCon cells ($1*10^6$) were intravenously injected into BALB/c mice. Seven days later, the animals were sacrificed, and their lungs were imaged using 2-photon microscopy (using 20x objective). 3D-reconstruction was performed using Imaris Bitplane® 7.2 Software, clearly showing the presence of tethers, interconnecting neighboring cells. Scale bar: 10 μm.

Supplementary Movie S10

GFP-expressing 4T1 cells (Con) or shEcad 4T1 cells (shEcad), and RFP expressing mouse embryo fibroblasts (MEF) were plated in two parallel compartments of a silicon insert (Ibidi®). When the cells within the two compartments reached confluence, the insert was removed, and the area between the two cell monolayers was imaged by live-cell video microscopy with 15 min intervals between frames, for a total of 66.75 hrs, using a 10X0.3NA objective. Scale bar: 10 μm.
